# Study protocol of Diabetes LEAP: a longitudinal study examining emotional problems in adolescents with type 1 diabetes and their parents/caregivers

**DOI:** 10.1186/s12887-019-1743-9

**Published:** 2019-10-24

**Authors:** Giesje Nefs, Linh Nguyen, Per Winterdijk, Esther Hartman, Theo Sas, Roos Nuboer, Ineke De Kruijff, Willie Bakker-van Waarde, Henk-Jan Aanstoot, Frans Pouwer

**Affiliations:** 1 Center of Research on Psychological and Somatic disorders (CoRPS), Department of Medical and Clinical Psychology, Tilburg University, PO Box 90153, 5000 LE Tilburg, The Netherlands; 2 Diabeter, National Treatment and Research Center for Children, Adolescents and Young Adults with type 1 diabetes, Blaak 6, 3011 TA Rotterdam, The Netherlands; 3 Radboud university medical center, Radboud Institute for Health Sciences, Department of Medical Psychology, Huispost 840, Postbus 9101, 6500 HB Nijmegen, The Netherlands; 4 Department of Pediatrics, Albert Schweitzer Hospital, Albert Schweitzerplaats 25, 3318 AT Dordrecht, The Netherlands; 5 Department of Pediatric Endocrinology, Erasmus Medical Center – Sophia Childrens’ Hospital, Rotterdam, The Netherlands; 6 Department of Pediatrics, Meander Medical Center, Maatweg 3, 3813 Amersfoort, TZ Netherlands; 7 Department of Pediatrics, Diabetes Centraal, St. Antonius Hospital, Soestwetering 1, 3543 AZ Utrecht, The Netherlands; 8 Department of Psychology, University of Southern Denmark, Campusvej 55, 5230 Odense M, Denmark; 9 School of Psychology, Deakin University, Locked Bag 20001, Geelong, VIC 3220 Australia; 10 STENO Diabetes Center Odense, Odense University Hospital, Odense, Denmark

**Keywords:** Type 1 diabetes, Adolescents, Parents, Depression, Anxiety, Longitudinal

## Abstract

**Background:**

Type 1 diabetes (T1D) is a chronic metabolic condition requiring intensive daily self-care to avoid both high and low blood glucose levels. Self-care and glycemic outcomes are particularly problematic in adolescence, a period known for its increased risk of emotional problems. However, the true scope of mood and anxiety disorders in adolescents with T1D is unknown. Earlier studies are limited by a small sample size, lack of diagnostic interview data, a focus on depression only, non-adolescent specific estimates, lack of information about parental emotional problems and/or a cross-sectional design. Diabetes LEAP is a two-year prospective observational cohort study examining (a) the prevalence and course of depression and anxiety in adolescents with T1D and their parents/caregivers, (b) the risk factors predicting the presence of these emotional problems, (c) their longitudinal relation with diabetes outcomes, and (d) the psychosocial care currently in place.

**Methods:**

Adolescents (12–18 years) from 8 Dutch pediatric diabetes clinics are interviewed using the DISC-IV to establish the presence of mood and anxiety disorders in the previous 4 weeks, the previous 12 months, and lifetime. They also complete questionnaires, including CDI-2, GAD-7, and PAID-T. Parents/caregivers complete PHQ-9, GAD-7, and PAID-PR. Follow-up assessments take place after 1 and 2 years.

**Discussion:**

This longitudinal study with diagnostic interviews in a large cohort of adolescents with T1D in the Netherlands will provide much needed information regarding the prevalence and course of depression and anxiety in this group, thereby opening avenues for proper recognition, prevention and timely treatment.

## Background

### Type 1 diabetes is an increasingly common and serious health problem

Type 1 diabetes (T1D) is among the most common chronic conditions in childhood, affecting more than one million children and adolescents worldwide and approximately 6000 young people in the Netherlands [1, 2]. It has been predicted that the number of young people with T1D will continue to increase [3, 4]. Having to deal with T1D can be challenging, not only for the children themselves but also for their families [5]. The Diabetes Control and Complications Trial has shown the importance of optimal management of blood glucose levels in the prevention of complications [6], but this can only be achieved with complex daily self-management. This includes blood glucose monitoring and injecting insulin multiple times a day or using an insulin pump, while taking into account factors such as carbohydrate intake and physical activity.

### Need for improved care and reduced psychosocial burden

Despite recent advancements in insulin-delivery and glucose-measurement technologies, glycemic outcomes remain suboptimal for the majority of young people with T1D, with less than one third achieving the international pediatric HbA_1c_ target (< 7.5%/ 58 mmol/mol) [7]. This will be even less when using the new target for children and adolescents of the International Society for Pediatric and Adolescent Diabetes (< 7% / 53 mmol/mol) [8]. It is well known that psychosocial problems are an important barrier for further improvements in diabetes care [5]. Psychosocial difficulties may interfere with the complex self-care activities and motivation of young people and their family, seriously impair diabetes management, and contribute to suboptimal glycemic outcomes [5]. In turn, suboptimal HbA_1c_ with concomitant high and fluctuating glucose values may impact psychosocial functioning. Thus, proper identification, diagnosis, and treatment of psychosocial issues and barriers are required to improve the diabetes care outcomes of young people with T1D. This is especially relevant for adolescents, as this group is particularly at risk for suboptimal glycemic outcomes [9, 10]. Reasons include hormonal changes, a mismatch between fully developed motor skills and ongoing cognitive maturity, conflicts between diabetes care and developmental tasks, as well as an increased vulnerability for the onset or worsening of emotional problems [11–13].

### The exact scope of mood and anxiety disorders in adolescents with T1D is unknown

Having to manage a demanding and rather unpredictable condition such as T1D can lead to significant distress, including feelings of depression and anger, worries about complications, and negative social interactions [14]. Young people with T1D also seem to have a 1.5 to 2 times increased risk of psychiatric conditions, including mood and anxiety disorders, compared to children without diabetes [15]. However, the exact size of the problem in adolescents is still unclear, as the available studies have several important methodological limitations. Most importantly, the majority of studies have relied on self-report questionnaires to assess symptoms of depression and anxiety, e.g. [16–18], rather than using a structured diagnostic interview to determine the presence of a psychiatric disorder. Much of what self-report scales assess appears to be (subclinical) diabetes-specific emotional distress rather than psychopathology [19]. The few studies that have used the diagnostic interview included less than 100 participants, did not distinguish between depression and anxiety, combined data from adolescents with data from children or young adults, or were conducted 20 years ago when diabetes treatment options were considerably different [20–25]. A recent Polish study among 207 children aged 8–18 years found that 4% had a current mood disorder and 16% had a current anxiety disorder [26]. However, this study might not be representative of the adolescent T1D population at large, as it excluded young people with significant co-existing diseases and did not distinguish estimates for children and adolescents separately [27].

### Information about the course of mood or anxiety disorders (and related care decisions) is scarce

As the majority of previous studies used a cross-sectional design, information with respect to the course of mood or anxiety disorders in adolescents with T1D is also scarce. Early data from small scale studies suggest that up to half of all young people with T1D will be confronted with a psychiatric disorder (for example a mood, anxiety, eating, or conduct disorder) at least once while growing up [20, 21]. Major depression appeared to be the most prevalent disorder, affecting approximately 28% of 92 American youth 10 years after the diagnosis of diabetes [21]. In addition, almost one fifth of this sample developed an anxiety disorder [21]. As a last limitation, studies that have determined the presence of a mood and/or anxiety disorder have not systematically assessed the care decisions that were subsequently taken. A high quality study is needed, establishing the prevalence and course of mood and anxiety disorders in a large cohort of adolescents with T1D, including their link with diabetes-specific distress, as well as a description of the treatment and follow-up that is provided.

### Parental factors might be a very important, yet understudied topic

Caring for an adolescent with T1D is challenging, e.g. due to differences in views on diabetes management and goals, and poses parents and caregivers at additional risk for emotional distress [28, 29]. Approximately 20–30% of parents of children with T1D report clinically significant distress, defined as life or parenting stress, anxiety or depression [29]. In the general population, parental emotional problems have been associated with increased risk of internalizing problems (such as anxiety or depression) in children [30–32]. In the context of T1D, parental emotional problems may not only have a direct negative effect on child mental health, but may also worsen diabetes management and glycemic outcomes [29, 33]. A continued level of parental involvement in diabetes care, adjusted to and stimulating the child’s self-efficacy beliefs, is one of the factors for successful diabetes outcomes in adolescence [34–37]. Emotional problems of parents may negatively interfere with their availability in diabetes care, either by under- or over involvement [38–41]. Even though parental emotional distress is common and has been associated with child distress and impaired diabetes management [21, 22, 29], it is infrequently included in (large) studies of children with T1D. Furthermore, longitudinal data on the course of these problems are currently lacking and emotional distress of parents/caregivers is not included as a standard risk factor in studies examining emotional problems in children with T1D.

## Methods/design

### Aim and design

Diabetes LEAP (Longitudinal study of Emotional problems in Adolescents with type 1 diabetes and their Parents) was designed as a two-year prospective study to determine the true scope of depression and anxiety in a large cohort of adolescents with T1D (12–18 years) and their parents/caregivers. Its aims are to:

1. Establish the prevalence and course of mood and anxiety disorders among adolescents with T1D and of elevated symptoms of depression and anxiety in parents/caregivers.
2. Describe treatment and follow-up, once the pediatrician has been informed of the presence of a mood or anxiety disorder.
3. Identify demographic, clinical and psychological risk factors associated with the occurrence of emotional problems in this group.
4. Examine the longitudinal relations of these problems with diabetes outcomes.

### Participants and procedure

Eligible adolescents with type 1 diabetes (12–18 years) and the parent/caregiver primarily involved in diabetes care are approached for participation. Exclusion criteria are diabetes duration of < 6 months, insufficient mastery of the Dutch language, moderate to severe intellectual disability, or severe psychosocial circumstances interfering with participation, all based on medical records and pediatricians’ judgement. Participants are recruited from 8 pediatric diabetes clinics across the Netherlands, including the Diabeter clinics (locations: Deventer, Groningen, Rotterdam, Schiphol, Veldhoven), Albert Schweitzer hospital Dordrecht, Meander Medical Center Amersfoort, and Diabetes Centraal (St. Antonius hospital) Utrecht.

Eligible families receive an information package about study participation, asking them to read the materials before their next usual care visit. During this visit, the pediatrician discusses the study with the adolescent and both of his/her parents. Families who agree to participate complete written informed consent. Adolescents can participate (with consent from parents/caregivers), even if the parent/caregiver him−/herself does not want to participate. The parent/caregiver cannot participate if the adolescent declines. At a subsequent visit, the adolescent undergoes a structured diagnostic interview to assess the presence and history of mood and anxiety disorders. During the same visit, he/she completes a questionnaire regarding (potential risk factors for) emotional problems. Parents/caregivers answer similar questionnaires, either at home or in the clinic. Pediatricians of the research team then report the presence of disorders to the adolescent’s pediatrician, who later completes a questionnaire about the course of action that is taken.

For both adolescents and parents/caregivers, follow-up assessments take place after 1 and 2 years. Participants who miss the time window for the one-year follow-up due to frequent rescheduling are considered as participants in the two-year follow-up with a missing one-year follow-up assessment. Participants who decline participation in the one- year follow-up are asked if they may be approached for the two-year follow-up. Assessments are preferably conducted face-to-face; if it is not possible to schedule a visit, interviews are held over the phone or via Skype.

The study protocol has been approved for all study sites by the Medical Ethics Committee of the Máxima Medical Center (NL48232.015.14). The first participant was included on 14-1-2015; recruitment and follow-up assessments are ongoing.

### Assessment of depression and anxiety in adolescents

At baseline and 1 and 2 year follow-up, the Dutch version of the Diagnostic Interview Schedule for Children (DISC-IV) [42] is used to establish the presence of mood and anxiety disorders in adolescents in the previous 4 weeks, in the previous year and during lifetime. The DISC-IV is a fully-structured diagnostic interview that can be used by trained lay interviewers to assess mental disorders based on the definitions of the Diagnostic and Statistical Manual for Mental Disorders Fourth edition (DSM-IV). Only the relevant modules of the DISC-IV are conducted. The introductory module assesses sociodemographic characteristics and includes the construction of timelines of the previous year and the whole life based on life-events. Module A (anxiety disorders) includes questions about social phobia, separation anxiety, specific phobia, panic, agoraphobia, generalized anxiety, selective mutism, obsessive-compulsive behavior and post-traumatic stress. We added questions assessing fear of hypoglycemia. Module C (mood disorders) contains questions about major depression, dysthymia, and (hypo)mania. At baseline, module L (Whole Life) is also conducted to assess lifetime prevalence of mood and anxiety disorders.

When emotional problems are established as being present in the previous 4 weeks of assessment, we examine the course of these problems at follow-up. Based on the timeline constructed in the introductory module, adolescents are asked to report periods of remission and/or recurrence if applicable.

At all measurement occasions, adolescents complete the Children’s Depression Inventory 2 (CDI-2) and the Generalized Anxiety Disorder 7-item scale (GAD-7) which assess the self-reported severity of depression and anxiety symptoms during the previous 2 weeks [43–45]. As an additional indicator of mood problems in the adolescent, the parent report version of the Children’s Depression Inventory 2 is used [43].

### Assessments of depression and anxiety in parents

At all three measurement occasions, parents/caregivers complete the Patient Health Questionnaire 9-item scale (PHQ-9) and Generalized Anxiety Disorder 7-item scale (GAD-7) to measure the severity of depression and anxiety symptoms in the previous 2 weeks [44, 46]. Both measures also have adequate psychometric properties to identify depression and anxiety disorders in adults (sensitivity 88 and 89%; specificity 88 and 82%, respectively) [44, 46].

### Assessment of treatment for depression and anxiety

In the case of a positive screening result, the pediatricians of the research team are notified and inform the doctor caring for the adolescent in question. The description of treatment and follow-up (once the pediatrician has been informed of the presence of a mood or anxiety disorder) include:

- Reason(s) for not making a referral, e.g. watchful waiting, current treatment, self-reported need for (additional) care, decisions/preferences of adolescent and his/her family, previous treatment.

- Institution/health care provider to which a referral is made, waiting list period, treatment provided, decisions/preferences of adolescent and his/her family.

### Assessment of other psychosocial and behavioral constructs

The Problem Areas in Diabetes Scale teen version (PAID-T) is used to assess diabetes-specific emotional distress in adolescents, which concerns negative emotions specifically related to living with diabetes and its self-management [47]. For parents, the Problem Areas in Diabetes Scale parent revised version (PAID-PR) is used [48]. Both adolescents and parents provide their perception of psychosocial problems during the previous 6 months (Strengths and Difficulties Questionnaire, SDQ, [49, 50]), following of diabetes care recommendations in the preceding month (Adherence in Diabetes Questionnaire, ADQ, [51]), and responsibility for different aspects of diabetes care (Diabetes Family Responsibility Questionnaire, DFRQ, [52]).

### Sociodemographic and clinical data

Medical records provide information regarding the adolescent’s gender, age, most recent weight and height, age at diabetes diagnosis, diabetes duration, physical/psychiatric comorbidities, insulin treatment modality (multiple daily self-injections or continuous subcutaneous insulin infusion), lifetime HbA_1c_ (i.e. composite of all HbA_1c_ values during treatment in the diabetes clinic), the mean HbA_1c_ in the past year and most recent HbA_1c_, glucose measurements and variability around the time of assessment (subsample only), diabetes-related hospitalisations, severe hypoglycemic events, and occurrence of diabetic ketoacidosis. Furthermore, the number of clinic visits, the number of other contacts (e.g. e-mail messages), cancelled visits, and no shows are extracted from medical records. Adolescents self-report their current educational level as part of the DISC-IV and physical/psychiatric comorbidities by completing a questionnaire. The questionnaire for parents includes items about their own gender, age, ethnic background, education, employment status, marital status, family socio-economic status, family composition, relation to the adolescent (e.g. biological parent, guardian), adolescent life-events, adolescent physical/psychiatric comorbidities, own history of mood/anxiety disorders, and current psychiatric problems in the family.

An overview of the measures included in diabetes LEAP is provided in Table 1

**Table 1:** Concepts and instruments used in Diabetes LEAP

Construct	Instrument	Baseline	1 year	2 year
*Adolescent*				
4-week (current) mood/anxiety disorder	DISC-IV	x	x	x
1-year history mood/anxiety disorder	DISC-IV	x	x	x
Lifetime history mood/anxiety disorder	DISC-IV	x		
Severity of depressive symptoms	CDI-2	x	x	x
Severity of anxiety symptoms	GAD-7	x	x	x
Diabetes-specific distress	PAID-T	x	x	x
Psychosocial problems	SDQ	x	x	x
Following diabetes-care recommendations	ADQ self-report	x	x	x
Responsibility diabetes care	DFRQ self-report	x	x	x
Care provided mood/anxiety disorder	Medical record, doctor	x	x	x
Gender, age, weight, height	Medical record	x		
Highest educational level	Medical record, self-report	x	x	x
Diabetes duration, age at diagnosis	Medical record	x		
Lifetime HbA_1c_	Medical record	x		
Past year and most recent HbA_1c_	Medical record	x	x	x
Glucose measurements/variability (subgroup)	Medical record	x	x	x
Physical/psychiatric co-morbidities	Medical record, self- & parent-report	x	x	x
Life-events	DISC-IV, parent-report	x	x	x
Insulin treatment modality	Medical record	x	x	x
Diabetes-related hospitalisations, severe hypoglycemic events, occurrence of diabetes ketoacidose	Medical record	x	x	x
Past year number of visits, other contacts, cancelled visits, no shows	Medical record	x	x	x
*Parents/caregivers*				
Elevated depressive symptoms	PHQ-9 ≥ 10	x	x	x
Elevated anxiety symptoms	GAD-7 ≥ 10	x	x	x
Diabetes-specific distress	PAID-PR	x	x	x
Adolescent depressive symptoms	CDI-2 parent-report	x	x	x
Psychosocial problems	SDQ parent-report	x	x	x
Following diabetes-care recommendations	ADQ parent-report	x	x	x
Responsibility diabetes care	DFRQ parent-report	x	x	x
Adolescent life-events	Parent-report	x	x	x
Gender, age, ethnic background, education, employment status, partner	Self-report	x	x	x
Socio-economic status family	Self-report, medical record	x	x	x
Primary diabetes caregiver	Self-report, doctor	x	x	x
Relation to child, description family	Self-report	x	x	x
Current and history mood/anxiety disorder	Self-report	x	x	x
Current psychiatric problems in family	Parent-report	x	x	x

### Ethical considerations

Informed consent is sought for (1) informing the pediatrician of study results in the case of a positive screen, (2) extracting clinical information from the medical record, and (3) using the data anonymously for scientific publications. The code of conduct issued by the national Central Committee on Research Involving Human Subjects (CCMO) relating to expressions of objection by minors participating in medical research is followed, as drafted by the Paediatric Association of The Netherlands (NVK). Adolescents are invited to enter a draw to win a tablet computer at each assessment round. Study visits are combined with usual care visits where possible; extra travel expenses are reimbursed.

### Data handling and storage

The handling of personal data complies with the Dutch Personal Data Protection Act. Families are assigned a unique study number (unique successive numbers, starting with 11001). This number is used as the subject identification code in the anonymous study data file. The file containing the link between research codes and specific families is saved on a secure server and is only be accessed by people conducting the assessments. Psychology students assisting in the data collection sign a research contract, based on the Dutch Personal Data Protection Act. The anonymized data set is saved on a secure server. Data will be stored at this server for at least 15 years after the end of the study, in accordance with Tilburg University regulations.

### Statistical analyses

Unless otherwise specified, statistical analyses will be performed using IBM SPSS Statistics v24, with significance level *p* < 0.05. The prevalence, course and treatment of emotional problems in adolescents with type 1 diabetes and their parents/caregivers (research aim #1 and #2) is established using descriptive statistics based on the sample at hand. Other analyses depend on the specific research questions and will be reported in detail in the papers concerned. These include logistic regression analysis (including a check of relevant assumptions) to examine which risk factors are associated with the occurrence of a mood and/or anxiety disorder (research aim #3) and longitudinal path modelling/structural equation modelling to examine the longitudinal relation between emotional problems and diabetes outcomes (research aim #4). To maximize the available data, the advanced Full Information Maximum Likelihood method will be applied to handle missing data. To get a sense of the required sample size, we posited a conservative logistic regression for the research question on risk factors for mood/anxiety disorders over the entire study period. As our study will be the first to determine the prevalence of these disorders in Dutch adolescents with type 1 diabetes, we calculated a range of sample sizes based on different cumulative event rates (175 to 400 required participants) [53]. Taking into account statistical restrictions [54], non-response and loss to follow-up, we have to approach all families from all participating centers who meet the inclusion criteria and none of the exclusion criteria (*n* = 831).

## Discussion

The proposed prospective observational study will provide a much needed indication of the prevalence, course, risk factors and clinical impact of emotional problems among adolescents with type 1 diabetes and their parents/caregivers, thereby opening avenues for proper recognition, prevention and timely treatment. This may alleviate stress on scarce health care resources, making way for policy adjustments and re-allocation of funds. Diabetes LEAP will also shed more light on the current trajectories of psychological care after detection of emotional problems. A better understanding of the decision-making process will facilitate development and adjustments towards optimal care. By disentangling psychopathology and subclinical diabetes-specific distress, we also hope to assist policy makers in obtaining a better match between the problems at hand and mental health professionals and interventions. Diabetes LEAP will also provide an important contribution to the scientific literature, allowing comparisons with data from other countries and sharing of lessons learned.

Some potential limitations must also be acknowledged. Recruitment and preservation of participants in research is often challenging, and even more so in the case of adolescents and sensitive topics [55]. Following previous recommendations to increase the response rate and reduce attrition, e.g. [56], we selected a research team with knowledge about diabetes and child psychology, involve paediatricians in the recruitment procedure, collaborate closely with site personnel, and offer proportional incentives for participation to families. To ensure sufficient power to conduct the longitudinal analyses, recruitment takes place in 8 paediatric diabetes clinics. However, sampling bias may occur if families experiencing emotional problems shy away from participation. Also, individuals who do not experience any problems might not feel inclined to participate. In an effort to minimize the risk of sampling bias and maximize the external validity of study results, the information package emphasized that participation in research is voluntary and that every adolescent/parent contribution is a valuable addition to the study. In order to limit attrition and support sustained commitment, we send participating families regular updates through clinic newsletters.

The data from this study will include results from psychiatric diagnostic interviews in a large cohort of adolescents with T1D in the Netherlands. We expect that it will further strengthen the scientific literature in this area, as we aim to provide much needed information regarding the scope of depression and anxiety in this group. This will hopefully contribute to better recognition, prevention and treatment of these problems and more optimal pediatric diabetes care.

## Data Availability

The datasets used and/or analyzed during the current study are available from the corresponding author on reasonable request.

## Abbreviations

ADQ: Adherence in diabetes questionnaire
CDI-2: Children’s depression inventory 2
DFRQ: Diabetes family responsibility questionnaire
Diabetes LEAP: Longitudinal study of emotional problems in adolescents with type 1 diabetes and their parents
DISC-IV: Diagnostic interview schedule for children
DSM-IV: Diagnostic and statistical manual for mental disorders fourth edition
GAD-7: Generalized anxiety disorder 7-item scale
NVK: Paediatric Association of The Netherlands
PAID-PR: Problem areas in diabetes scale parent revised version
PAID-T: Problem areas in diabetes scale teen version
PHQ-9: Patient health questionnaire 9-item scale
SDQ: Strengths and difficulties questionnaire
T1D: Type 1 diabetes
